# Head holder and cranial window design for sequential magnetic resonance imaging and optical imaging in awake mice

**DOI:** 10.3389/fnins.2022.926828

**Published:** 2022-08-16

**Authors:** Signe H. Mikkelsen, Boris Wied, Vitalii Dashkovskyi, Thomas Beck Lindhardt, Lydiane Hirschler, Jan M. Warnking, Emmanuel L. Barbier, Dmitry Postnov, Brian Hansen, Eugenio Gutiérrez-Jiménez

**Affiliations:** ^1^Center of Functionally Integrative Neuroscience, Aarhus University, Aarhus, Denmark; ^2^Leiden University Medical Center, Leiden, Netherlands; ^3^Univ. Grenoble Alpes, Inserm, U1216, GIN, Grenoble Institut des Neurosciences, La Tronche, France

**Keywords:** multimodality, interpretation, cranial window, restraint design, surgical protocol, awake imaging

## Abstract

Medical imaging techniques are widely used in preclinical research as diagnostic tools to detect physiological abnormalities and assess the progression of neurovascular disease in animal models. Despite the wealth of imaging options in magnetic resonance imaging (MRI), interpretation of imaging-derived parameters regarding underlying tissue properties is difficult due to technical limitations or lack of parameter specificity. To address the challenge of interpretation, we present an animal preparation protocol to achieve quantitative measures from both MRI and advanced optical techniques, including laser speckle contrast imaging and two-photon microscopy, in murine models. In this manner, non-translatable methods support and improve interpretation of less specific, translatable methods, i.e., MRI. Combining modalities for improved clinical interpretation involves satisfying the requirements of various methods. Furthermore, physiology unperturbed by anesthetics is a prerequisite for the strategy to succeed. Awake animal imaging with restraint provides an alternative to anesthesia and facilitates translatability of cerebral measurements. The method outlines design requirements for the setup and a corresponding reproducible surgical procedure for implanting a 3D printed head holder and cranial window to enable repeated multimodal imaging. We document the development, application, and validation of the method and provide examples confirming the usefulness of the design in acquiring high quality data from multiple modalities for quantification of a wide range of metrics of cerebral physiology in the same animal. The method contributes to preclinical small animal imaging, enabling sequential imaging of previously mutually exclusive techniques.

## Introduction

Imaging techniques are widely used to investigate neural function in animals and humans. Preclinical *in vivo* examinations of brain hemodynamics provide valuable information to understand pathophysiology associated with neurovascular diseases. Such information can be used to identify potential biomarkers and pre-symptomatic indicators of physiological abnormalities to implement treatment and preventative strategies (GBD Neurology Collaborators, 2019). Magnetic resonance imaging (MRI) has become a staple non-invasive macroscopic imaging technique in most clinical research facilities, useful in providing anatomical images, blood oxygen level-dependent signals (BOLD) through functional activation, and perfusion measurements, among others (Kasban et al., 2015). Despite its many applications, technical limitations and lack of parameter specificity of MRI challenge the interpretation of parameters regarding underlying tissue properties. Nevertheless, MRI is established as a relevant and translatable tool in preclinical research, as *in vivo* studies in murine models enable investigations of physiological properties and functional changes that would otherwise be infeasible in humans.

One strategy to address the challenge of interpretation is to combine MRI with optical imaging modalities, which offer higher resolution and more specific measures of cerebral physiology (Desjardins et al., 2019). Though high-resolution optical imaging modalities are non-translatable to humans, they provide explanations for underlying tissue properties (MightEx, n.d.; Sicard et al., 2010) to support and improve the interpretation of MRI. While the use of animals in preclinical studies of cerebral hemodynamics cannot be avoided, integrating different techniques in a single animal for a multimodal imaging approach reduces the number of animals needed in a study (Hooijmans et al., 2010; Hohlbaum et al., 2017). The animal must be still during acquisition for MRI and optical imaging to succeed. Therefore, small animal imaging is typically performed with anesthetics to achieve immobilization and remove motion artifacts causing image distortion and reduced image quality. Although anesthetic agents such as isoflurane have fast induction- and recovery rates and minimal long-term side effects (Haensel and Martin, 2015; Munting et al., 2019), the use of anesthetics during imaging poses the risk of decreased signal intensity, as physiological functions such as overall brain activity, cerebral hemodynamic response, and oxygen metabolism are suppressed (Haensel and Martin, 2015; Li et al., 2018). A restraint setup to prevent the movement of awake mice removes the need for anesthetics, eliminating unwanted side-effects during imaging.

This article presents a design for awake-restrained imaging and a corresponding surgical procedure for sequential MRI and optical imaging in mice. Surgical procedures for cranial window and head holder implantation are combined in a preparation suitable for both acute and longitudinal studies. A cranial window for *in vivo* optical imaging serves the purpose of accessing the brain while maintaining normal brain conditions for repeated imaging (Yang et al., 2015; Heo et al., 2016; Silasi et al., 2016), and a 3D printed restraint consists of two head pieces, an imaging bed for MRI, and an imaging bed for optics to provide stability of the neck and head of the mouse. The preparation for chronic cranial window and head holder implantation is applicable for MRI and various optical imaging techniques. The effectivity of the design is demonstrated here using pseudo-continuous arterial spin labeling (pCASL), laser speckle contrast imaging (LSCI), optical intrinsic signal imaging (OISI), and two-photon microscopy (TPM). To validate the imaging setup, anatomical images and parameters from pCASL to estimate cerebral blood flow (CBF) were acquired with MRI, and changes in hemodynamic response during whisker stimulation (relative oxygenated and deoxygenated hemoglobin (HbO/HbR) and relative blood flow index) were obtained with simultaneous OISI/LSCI and TPM.

## Materials and equipment

Materials for surgical preparation of chronic cranial window and head holder implantation and corresponding imaging setup.

### Surgical instruments

∘ Stereotaxic frame with arm (KOPF Instruments, Tujunga, CA, United States)
∘ Iris scissors (F.S.T., GmbH, Germany)
∘ 2 McPherson Forceps (Angled 45 degrees, smooth; F.S.T., GmbH, Germany)
∘ 1 Dumont #3 Forceps (F.S.T., GmbH, Germany)
∘ Scalpel holder (F.S.T., GmbH, Germany)
∘ Carbon steel scalpel blade (Swann Morton, Sheffield, England)
∘ Surgical spatula (F.S.T., GmbH, Germany)
∘ 0.5 mm drill burr (HM71 005, Hager and Meisinger GgmH, Germany)

### General materials

∘ Heating pad with homeothermic feedback control
∘ Electric animal trimmer (Isis, Aesculap)
∘ Disposable surgical cover
∘ Cotton buds
∘ Toothpicks
∘ Parafilm
∘ Hemostatic sponge (Option: Spongostan, Ethicon, Spongostan Dental, 1 cm × 1 cm × 1 cm, MS0005)
∘ Tissue paper
∘ Weighing boat
∘ Lens paper
∘ Silicone mixing cup
∘ 1 ml pipette
∘ 5 ml × 1 ml syringes with 30 G needles (or preferably insulin syringes with needle)

### Medication

∘ Isoflurane (3% induction and 1.75% for surgical anesthesia)
∘ Temgesic (Buprenorphine) (2 μl/g) – painkiller
∘ Xylocaine (10 mg/ml) – local anesthesia
∘ Carprofen (10 mg/kg) – anti-inflammatory
∘ Ampicillin (200 mg/kg) – antibiotic
∘ Dexamethasone (4.8 mg/kg) – edema reduction
∘ Isotonic glucose – hydration

### Solutions

∘ Ethanol (70% denatured)
∘ Acetone
∘ Pressurized air
∘ Sterilized 0.9% NaCl solution
∘ Hair removal cream (Option: Veet, Reckitt Benckiser Health Care, United Kingdom)
∘ Ophthalmic ointment (Viscotears 2 mg/g, 10 g)
∘ Superglue (Loctite powerflex gel, 3 g, Henkel)
∘ VetBond tissue adhesive (3 ml, 3 M, Saint Paul, MN, United States)
∘ Meliodent Rapid Repair Powder (Kulzer GmbH, Hanau, Germany)
∘ Meliodent Rapid Repair Liquid 0.5 L (Forstec Dental AB, Malmö, Sweden)
∘ Kwik-Sil (World Precision Instruments, FL, United States)
∘ Optical adhesive (NOA61, Norland Products, NJ, United States)

### Restraint and cranial window

∘ 3 mm × 3 mm Ø microscope cover glasses (41001103, Glaswarenfabrik Karl Hecht GmbH and Co., Germany)
∘ 1 mm × 5 mm Ø microscope cover glass (41001105, Glaswarenfabrik Karl Hecht GmbH and Co., Germany)
∘ 3D printed frontal head piece
∘ 3D printed collar head piece
∘ 3D printed MRI bed
∘ 3D printed optical imaging bed
∘ 3D printed lid for optical imaging bed
∘ Canvas fabric

*All 3D printed designs can be downloaded from our GitHub repository.^1^

### Machinery and misc

∘ Ultrasonic cleaner (Model Y-009, 1.3 L, 60 W, RS Components, Corby, United Kingdom)
∘ UV light-curing box (Mega Electronics, Cambridge, England)
∘ Fume hood
∘ Foredom Micromotor Control drill (Foredom Electric Company, Bethel, CT, United States)
∘ Steri 250 bead sterilizer (Simon Keller Ltd., Switzerland, 220–240 V, 50 Hz, 90 W)
∘ Ohropax Classic Ear plugs (Orhopax, Persano Group A/S, Graested, Denmark)
∘ Imaging stage:
  ∘ Hook and loop fastener
  ∘ M4 thumb screw
  ∘ M6 metal screws
  ∘ M2 plastic screws
  ∘ M2 screw threader
  ∘ Tubing for air puff
  ∘ Stage parts:
    • MS1.5R – Mini-Series Optical Post, Ø6, *L* = 1.5? (Thorlabs)
    • MS1.5R/M – Mini-Series Optical Post, Ø6, *L* = 38 mm (Thorlabs)
    • SA2 Series (Newport, Irvine, CA, United States)
    • M-MCA-1 – Right-Angle Post Clamp (Newport, Irvine, CA, United States)
    • MSPH-2 – Optical Post Holder (Newport, Irvine, CA, United States)
    • MSPH-1 – Optical Post Holder (Newport, Irvine, CA, United States)
    • Optical Breadboard Plate, 150 mm × 150 mm, M6 (Newport, Irvine, CA, United States)

## Methods

The method aims to present and validate a design for restraint and a corresponding surgical procedure for integrated awake imaging with MRI and optical techniques. Here, we describe a procedure which offers unobstructed imaging of a small area of the cortex, is based on the success of the design of the imaging beds, and is compatible with MRI. In addition, design requirements for stability and mouse comfort in imaging beds and head holders are described.

### Design

#### Restraint

**FIGURE 1:**
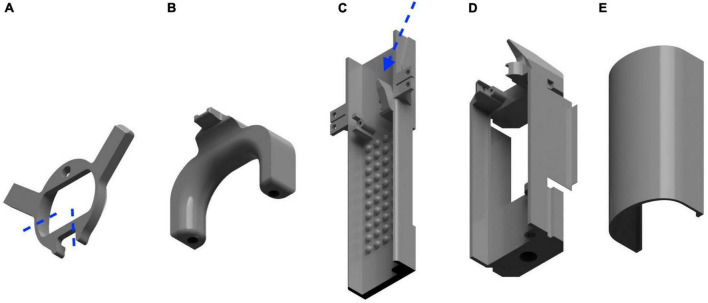
3D designs for awake-restrained imaging. **(A)** Frontal head holder with markers (blue) for cutting to allow space for the collar; **(B)** Collar head holder for neck stabilization. Holes in the base are threaded prior to surgery for fixation to the bed using plastic screws; **(C)** MRI imaging bed with air-puff applicator (blue arrow); **(D)** Optical imaging bed; **(E)** Lid for optical imaging bed.

Magnetic resonance imaging compatibility is provided with 3D printed restraint. All 3D constructions for restraint were designed using Autodesk Fusion 360 software and printed in polyethylene terephthalate glycol (PETG) material on a commercial 3D printer (Prusa I3 MK3S+; Ultimaker B.V., Utrecht, Germany). Five restraint components are included in the setup: frontal head piece, collar head piece, MRI bed, optical imaging bed, and lid for optical imaging bed (Figure 1). The collar head piece and MRI bed were modified from Han et al. (2019).

The frontal head piece (Figure 1A) provides stability of the anterior region of the head during optical imaging sessions. The frontal head piece is based on and modified from a head holder described by Kilic et al. (2020). Two protrusions extending from the circular frontal head piece allow fixation to the imaging stage. Since TPM requires water immersion objectives with short working distances, the posterior part of the frontal head piece is removed (see Figure 1A) to ensure space for the objective and avoid a high dental acrylic build when placing the collar. The collar head piece (Figure 1B) consists of an anterior projection for attachment onto the occipital bone and a collar-like component to stabilize the neck during MRI scans. Two holes in the base of the collar, threaded prior to surgical preparation, are used to secure the collar to the MRI bed with M2 plastic screws. The MRI bed (Figure 1C) is equipped with low walls to contain the mouse, extruding gripping points on the floor of the bed to prevent gliding on the slippery plastic surface, and an angled air-puff applicator for stimulation of the sensorimotor cortex. The mouse is further stabilized during scanning with plastic screws securing the bed to the bore insert of the MRI scanner. A comparable bed suitable for optical imaging (Figure 1D) consists of a fabric cradle for comfort and attachment of an accelerometer for movement detection, a sliding lid (Figure 1E), an angled air-puff applicator for stimulation, a hole for a metal rod insert enabling rotation of the bed, and screw holes to fasten the bed to the rod of the imaging stage. Rotational ability of the bed maintains the natural position of the mouse while imaging various areas of the brain. For sanitation purposes, the fabric can be attached/detached with hook and loop fastener and washed between mice. The same fastener is further used for attachment of an accelerometer below the body of the mouse. The angled air-puff design enables coupling of an air-puff tube for whisker stimulation and ensures a reproducible area of stimulation. Features of the imaging beds were designed to provide maximum comfort for the mouse during restraint to maintain their natural body position. All restraint designs can be downloaded from our GitHub repository (see text footnote 1).

#### Mechanics

**FIGURE 2:**
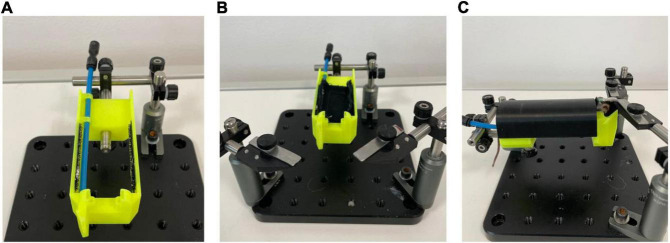
Imaging stage setup for optical imaging. **(A)** Stage and metal rod for raising the bed. An air-puff tube delivers air-puffs through an angled stimulator at the front of the bed; **(B)** Fabric attached to the bed using hook and loop fastener to increase mouse comfort; **(C)** Mouse fixed in the imaging bed using clamps on the frontal head holder. A lid ensures minimal movement without restricting breathing.

The imaging stage for optics (Figures 2A–C) is designed with bed rotation and head holder fixation in mind. Optical post holders (MSPH-1, MSPH-2), post clamps (M-MCA-1), and optical posts (MS1.5R, MS1.5R/M) are used to raise the bed on an aluminum breadboard plate. The bed is fastened to the metal rod with an M4 thumb screw. Post holders are attached to the bed using M6 screws. The head of the mouse is fixed with two clamps on the frontal head holder (Figures 2B,C). A removable fabric cradle is attached to the bed using hook and loop fastener (glued to the bed and sewn onto the fabric). Another piece of hook and loop fastener is sewn onto the underside of the cradle to attach an accelerometer. Blue tubing is used for delivery of air puffs. The MRI bed is inserted into the MRI bore and does not require a stage.

#### Windows

We used a crystal plug for cranial window preparations as described in Desjardins et al. (2019). A plug to fill the craniotomy and prevent infection and bone regrowth was created with three 3 mm Ø glasses and one 5 mm Ø glass. A stack of 3 mm Ø glasses was prepared using a needle to place a small drop of Norland optical adhesive onto a 3 mm Ø glass and forceps to place another glass on top. This step was repeated with the remaining 3 mm Ø glass. A small drop of adhesive was then placed onto a 5 mm Ø glass, and the stack of 3 mm Ø glasses was centered on top. The windows were cured in a UV light box for 60 s.

### Experimental paradigm

The following procedure provides a standard surgical protocol for a chronic cranial window and head holder implantation for awake-restrained imaging with MRI and optical imaging techniques. The procedure combines previously described surgical techniques for MRI awake-restrained imaging (Lindhardt et al., 2022, parallel study) and serial awake optical imaging (Desjardins et al., 2019; Kilic et al., 2020).

#### Experimental animals

All procedures were conducted in accordance with the Danish Animal Experiments Inspectorate under the permit number 2017-15-0201-01241.

For this protocol, C57BL/6 mice (male and female) were housed in conditions of 12 h light/dark cycle, 22–24°C, 55 ± 10% humidity, and *ad libitum* feeding. Mice were socialized before and after surgery to reduce mouse timidness and ease handling (King et al., 2005; Prescott and Lidster, 2017).

#### Medication

Medication was administered based on dilution and mouse weight, except for xylocaine and isoflurane. Dexamethasone (4.8 mg/kg, stock 4 mg/ml) was administered intraperitoneally (IP) 1 day prior to surgery to reduce brain edema due to removal of the skull. Anesthesia was induced with 3% Isoflurane (with 100% O_2_ in 0.4–1 L/min, depending on mouse size) and then maintained at 1.75% for the duration of the surgical procedure. Before incision, Xylocaine (10 mg/ml, stock 10 mg/ml) was administered subcutaneously (SQ) in the surgical region as local anesthesia. IP injections of antibiotic (Ampicillin 200 mg/kg, stock 1 g of 2.7 mmol Na), anti-inflammatory (Carprofen 10 mg/kg, stock 50 mg/ml), and painkiller (Temgesic 0.2 μl/g, stock 0.3 mg/ml) were also administered during the surgical procedure and post-operative period.

#### Surgical preparation

Approximate surgical time: 1 h 30 min

**FIGURE 3:**
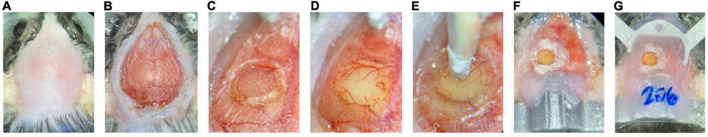
Surgical steps. **(A)** Mouse fixed in stereotaxic frame with a cleared surgical site; **(B)** Droplet-shaped removal of the skin to expose the skull and Vetbond tissue adhesive applied to the edges of the scored skull; **(C)** Drilling of 3 mm cranial window; **(D)** Bone flap removed to expose the brain; **(E)** Glass plug placed, toothpick lowered, and glue applied to window edges. Potential bleeding below the silicone will reabsorb within a few days; **(F)** Meliodent applied in multiple components to secure the window, the collar head piece, and the frontal head piece; **(G)** Chronic cranial window, frontal head holder, and collar head holder fixed to the skull with Meliodent.

See Figure 3 for the placement of a 3 mm Ø cranial window on the left hemisphere.

##### Pre-operative preparation

- Handle the mice for a minimum of 5 days prior to surgical preparation to reduce stress, improve recovery, and ease handling during restraining sessions.
- Administer dexamethasone, according to weight, 1 day prior to surgery to reduce inflammation (edema).
- Prepare the head holders: use a scalpel to scratch the underside of the head holders (collar and headpiece), and cut the lower left part of the frontal head piece to make room for the TPM objective.
- Prepare plugs with three 3 mm Ø and one 5 mm Ø circular glass coverslips for a total of four coverslips. *Note:* It is recommended to clean the cover glass before preparing the glass plugs. To clean the windows, place the glass into a crystal vial with acetone and sonicate for ∼15 min. In a fume hood, remove the windows from acetone, rinse with ethanol, and clean with lens paper.

##### Surgical procedure

1. Sterilize surgical instruments in a bead sterilizer, and clean all surgical surfaces with 70% denatured ethanol.
2. Induction of anesthesia is performed in an induction chamber with 3% isoflurane in 100% oxygen flow. Once sedated, reduce isoflurane concentration to 1.5–2% isoflurane, and fix the mouse in a stereotaxic frame equipped with a mask for gas anesthesia. Use a heating pad with a homoeothermic temperature controller to maintain body temperature at 37°C. *Note:* Routinely monitor respiration rate and reflexes throughout surgery to determine the depth of anesthesia as well cardiac and respiratory functions. Adjust Isoflurane dose as necessary.
3. Remove fur on the head and neck of the mouse using an electric animal shaver. Briefly apply hair removal cream to the skin and remove using cotton swabs. *Note:* Extended exposure to hair removal cream to the skin may cause chemical burns. Therefore, it is recommended to clean the scalp with gauze wet with sterile saline.
4. Clean the scalp with ethanol, apply ophthalmic ointment to the eyes to protect them from corneal drying, and subcutaneously administer xylocaine at four points uniformly distributed along the edge of the skull.
5. Administer Temgesic, Carprofen, and Ampicillin IP. *Note:* Temgesic should be administered twenty minutes before incision to ensure effective pain coverage.
6. At the posterior edge of the occipital bone, make a small incision with scissors, and cut the skin in a droplet shape to expose the skull. Remove and push connective tissue toward the lateral border of the skull (temporal suture region). Scrape the periosteum with forceps and a scalpel, then clean the skull with saline. Cut a small part of the neck muscle from the occipital bone to allow space to attach the head holder. *Note:* Use small pieces of saline-soaked hemostatic sponge to stop potential bleedings caused by removing the muscle.
7. Using a toothpick or needle, apply Vetbond tissue adhesive to the skin around the skull to keep it in place. *Note:* Use forceps to slightly lift the skin to apply the adhesive between the skin and the tissue. It is essential to allow the glue to harden completely to avoid forming a non-adhesive membrane when in contact with water. Non-toxic superglue (Loctite precision glue) can be used as an alternative.
8. Scratch the occipital bone using a scalpel to increase surface area. *Note:* Scratch at various angles to cover as much of the bone as possible. Take care when scratching over the sutures due to the risk of bleeding.
9. For imaging of the somatosensory area of the barrel cortex, we recommend the center of the window be placed onto the center of the whisker C2 (1.5–2 mm anterior-posterior, 3 mm medio-lateral to Bregma). Apply water as a coolant while drilling to prevent overheating the brain tissue. Use a diamond grinding bit and a micromotor drill to drill a ∼3 mm circle until the skull is soft. Place a saline-soaked hemostatic sponge to stop any active bleeding and further soften the bone to make removal easier. Use forceps to gently press on the drilled area to test if the bone is thin enough and ready for removal. The skull is ready to be removed when it depresses easily when pressed. Carefully insert sharp forceps (Recommended: Dumont #3) just under the raised skull in the center of the thinned area to loosen the bone, and flip the piece of skull to expose the brain. Use a hemostatic sponge to remove any bone dust from drilling and stop bleedings from the brain. *Note:* Bone regrowth may occur from bone particles. Therefore, it is recommended to discard each piece of hemostatic sponge after use to avoid spreading particles onto the brain. Keep the brain moist with a hemostatic sponge while preparing for window placement. No active bleeding should occur at the moment of closure as it may induce bone regrowth or inflammation.
10. Fix a toothpick to the arm of the stereotaxic frame. The toothpick is intended to hold the glass while waiting for the glue to dry. Do not lower until the window is placed.
11. Use a small needle or a toothpick to mix Kwik-Sil, and place a small drop onto the exposed brain to create a barrier between the glass and the brain. *Note:* Small bleedings below the silicone will usually reabsorb within a matter of days. This protocol uses non-toxic silicone below the window, but similar results can be achieved without using silicone.
12. Carefully clean the window with lens paper, and place the window with the 3 mm glasses facing downward to create a plug (window flat side up). Lower the toothpick to keep the glass in place. Cut away excess Kwik-Sil using a scalpel and forceps. Use a small needle to apply gel cyanoacrylate glue (Loctite) to fix the window to the skull; apply it to the skull, and pull it onto the outer part of the window. Ensure the glue has hardened before removing the toothpick holding the glass (∼15 min). *Note:* Forceps should be used to support the window until the toothpick is lowered. Gel cyanoacrylate glue (Loctite) typically takes approximately 10 min to harden completely.
13. Apply cyanoacrylate precision glue (Loctite) to cover the scratched area of the skull to create a non-toxic, protective layer between the skull/tissue and the dental cement. A thin cyanoacrylate superglue (Loctite) layer will harden within approximately 5 min.
14. Place the forearms beside the body, and position the collar of the head holder on the neck.
15. Prepare a semifluid mixture of dental acrylic (Meliodent) in a silicone mixing cup. Apply a thin layer of dental acrylic around the edge of the window, covering the hardened glue and part of the skull. Next, apply dental acrylic to the occipital bone. Quickly push the protruding part of the collar into the mixture and hold it in position within the area of the occipital bone until hardened. *Note:* A small spatula can be used to mix and apply dental acrylic. Complete hardening of the acrylic mixture may take up to 15 min, but the collar can be released once partially hardened (3–5 min). Make multiple components of dental cement to strengthen the preparation by spreading the force of movement on the head piece and collar. Components made in this preparation include securing the window, fastening the collar, fastening the front head piece, and connecting all pieces.
16. Center the head piece over the frontal bones, tilted slightly to the left (window on the left side), and secure with a drop of glue. Once properly positioned, apply dental acrylic on the frontal bones, along the right side of the skull. Place enough cement to stabilize the holders, but leave space for the objective. *Note:* Due to space constraints in the MRI bore, ensure the head holder is not angled upward. The hardened resin can be thinned by drilling if there is not enough space for the objective. A high dental acrylic build will be an obstacle for scanning.
17. Apply dental acrylic to connect the head holders to strengthen the adhesion and seal the surgical site. Once hardened, mark an ID on the acrylic, and cover it with a thin layer of resin.
18. Stop isoflurane flow and remove the mouse from the stereotaxic frame. Allow it to remain on the heating pad or move it to a recovery chamber.
19. Weigh the mouse for initial post-surgery weight.

##### Post-operative care

1. Place the mouse in a 37°C recovery chamber until the anesthesia wears off and the mouse appears stable and with voluntary movement.
2. Place soft food (powder food + water in a small weighing boat) at the bottom of the home cage once the mouse is returned to the cage. *Note:* If the operated mouse was housed with many non-operated mice, consider housing it separately post-surgery. Fighting between mice may occur (particularly among male mice), and fighting does not permit the operated mouse proper recovery conditions (food and rest).
3. Administer medication (Ampicillin, Carprofen, Temgesic, 50 μl saline, 50 μl isotonic glucose) IP. Give soft food for four post-operative days. *Note:* Temgesic can be given in water (2 ml bup. in 120 ml H_2_O) if mice are individually housed. However, consider that Temgesic might change the water flavor and discourage proper hydration. Replace the soft food daily.

##### Human termination criteria

In cases where more than 25% of initial body weight was lost, excessive pain or infection was evident, and/or the surgical procedure was unable to be fully executed, experimentation was abruptly ceased by euthanasia of the animal with an overdose of pentobarbital (recommended 500 mg/kg).

#### Placement of catheter for imaging

For administration of dyes and drugs during imaging, a venous catheter can be placed in the tail of the mouse. For this procedure, the mouse was anesthetized (induced with 3% Isoflurane in 100% oxygen and maintained with 1.5–2% Isoflurane in 100% oxygen using a mask) to place the catheter. The catheter was fixed with dental acrylic before injection. It is recommended to warm up any administered fluid to body temperature to avoid pain during injection. The mouse was positioned in the optical imaging bed during anesthesia waning and regained consciousness while restrained.

### Imaging

**FIGURE 4:**
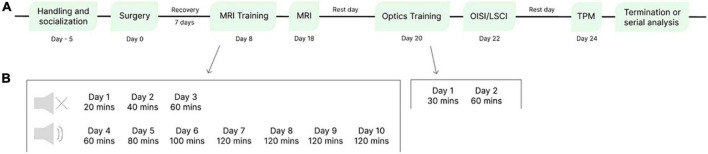
Experimental timeline. **(A)** Timeline from handling and socialization of mice to experiment termination. Surgery per animal was approximately 1 h and 30 min, training consisted of 10 days of restraint acclimation, followed by image acquisition with MRI, 2 days of optics training, OISI/LSCI, and TPM. Following image acquisition, the experiment can be terminated or continued as a longitudinal study. **(B)** Training protocols for MRI and optical imaging. MRI training days 1–3 were conducted without MRI noise.

Following a recovery period, the mice were trained in a mock MRI environment for acclimation to restraint in preparation for acquiring parameters from MRI to limit severe motion artifacts during imaging (Hurst and West, 2010; Dunn, 2014). Sample data were acquired from MRI, LSCI/OISI, and TPM. See Figure 4 for the experimental timeline.

#### Habituation to restraint

The protocol for sequential imaging was designed based on training protocols for optical imaging (Desjardins et al., 2019) and *in vivo* awake MRI imaging (Lindhardt et al., 2022). All mice were habituated to restraint in the beds and further trained for acclimation to MRI and optical imaging environments prior to scanning sessions. MRI training occurred in a mock MRI environment, as described in Lindhardt et al. (2022), over 10 days, starting with 20 min of restraint and increasing restraint time by 20-min increments daily until 2 h. A mock MRI environment consisted of a 3D printed box, MRI bed compartments, and speakers for transmitting MRI sound recordings of the actual sounds produced from the MRI sequences. Sounds were transmitted *via* digital audio software (Audacity, Audacity Team), and mice were equipped with earplugs (Ohropax, Graested, Denmark) for training days with MRI sound. Mice can be placed into the beds with or without anesthesia; well-handled mice are easily fixed while awake. When placing the mouse in the bed, the neck skin was gently pulled back to avoid skin pinching between the collar and the bed. The MRI scan was performed on day 18 after surgery, and after one rest day, training for optical imaging was conducted over 2 days (Day 20: 30 min, Day 21: 60 min) using the setup for optical imaging (Figures 2, 4).

#### *In vivo* magnetic resonance imaging and analysis

Experiments were performed on a 20 cm bore size Bruker BioSpec 9.4T (Avance III) small animal MRI system equipped with a 76 mm quadrature transmitting volume coil and a four-element rodent brain surface array cryoprobe for reception. BGA-12HP gradients capable of 650 mT/m were used (Bruker Biospin, Ettlingen, Germany). The mice were restrained in the MRI bed, equipped with ear plugs, and attached to the bore insert using plastic screws. A cushioned sensor between the mouse and the bed monitored respiration rate, and a layer of Kwik-Cast was applied onto the window and implant to protect the surfaces and reduce the amount of air between the skull and the coil. The Kwik-Cast was easily removed after scanning and did not affect the integrity of the window. Localizer scans (FLASH) were acquired to determine head positioning in the coronal, sagittal, and axial slice planes. A pCASL program package with parameters based on a prior protocol (Hirschler et al., 2018) was applied for acquisition of echo planar images (EPI) and parameters required for calculation of CBF. Longitudinal relaxation values were mapped using data acquired with Bruker’s T1_T2map_TrueFISP sequence. Scan parameters were TE/TR = 2.2/4.4 ms, 8 segments, TrueFISP SSFP mode, flip angle 60°, scan repetition time 18 s. A total of 60 inversion times were used ranging from 90 ms (minimum) to 8.4 s. An in-plane resolution of 220 μm × 220 μm was used. Total scan time was 2 min and 24 s. T1 was estimated using in-house MATLAB scripts, and the obtained T1 values were in agreement with those reported in rodent brain at our field strength (9.4T) (de Graaf et al., 2006).

Total scan time was approximately 37 min, and scanning and software preparation were completed within the time allotted for training. Image quality was assessed from modality specific requirements including signal to noise ratio, geometry [sharpness and geometrical distortions (correct shape)], presence of various distortions caused by noise or motion artifacts (Krupa and Bekiesińska-Figatowska, 2015), and acquisition of data similar to studies without the combined setup.

#### *In vivo* optical intrinsic signal imaging/laser speckle contrast imaging and analysis

To acquire multispectral and speckle images, we used an integrated OISI and LSCI system as previously described in Sunil et al. (2020). The system is equipped with two CMOS cameras: ORCA-Flash4.0 V3 (Hamamatsu) for OISI and acA2040-90 μm USB 3.0 (Basler) for LSCI. Images were acquired using a NIR 5× objective with 0.14NA and 37.5 mm working distance (W.D. – Mitutoyo, Kawasaki, Japan).

The OISI system sequentially illuminated the cortex with three mounted light-emitting diodes (LED) centered at 470 nm (M470L5; Thorlabs, Newton, NJ, United States), 530 nm (M530L4; Thorlabs), and 625 nm (M625L4; Thorlabs). The beams were collimated and filtered around the center wavelength. The LEDs synchronize with a transformer receiving a 5V signal from the camera to activate the RBG light beams. LED trigger mode enables the camera to determine the switch between colors depending on the desired frame rate. The blue LED (470 nm) was turned off and the corresponding frames were discarded during the scanning sessions. For OISI, the CMOS camera was triggered using HCImage software (Hamamatsu Photonics K.K., Hamamatsu, Japan) *via* an external trigger to synchronize with an accelerometer and to trigger the air puff stimulator (Powerlab, National Instruments, Austin, TX, United States). Images of 512 × 512 pixels (6.5 μm^2^ pixel size) were acquired at 30 fps with 20 ms exposure. OISI images were processed with a package to estimate oxy- (HbO) and deoxyhemoglobin (HbR) (Sunil et al., 2020).

For illumination of the entire window for LSCI, we used a 785 nm laser diode (LPC-785-FC, Thorlabs) controlled by a power unit (LDC205C, Thorlabs). Prior to detection, emitted light was split by a beam splitter (FF640-FDi02-t3, Seamrock, Rochester, NY, United States), and a 785 nm bandpass filter (Thorlabs) was used to filter the laser speckle signal to prevent signal bleed-through. A custom design software was used for LSCI recording, as previously described in Postnov et al. (2016). Images of 1024 × 1024 pixels (5.5 μm^2^ pixel size) were acquired at 100 fps with 5000 μs exposure. Additionally, a dedicated package (Postnov et al., 2016; Sunil et al., 2020) was used to perform contrast analysis and estimate relative blood flow index (rBFI).

#### *In vivo* two-photon microscopy and analysis

A load of 200 μl of 5% (5 ml/ml) solution of Texas-Red dextran 70 KDa was administered *via* a tail catheter. TPM imaging was performed using an Ultima-IV two-photon system (Bruker Corporation, Billerica, MA, United States) and PrairieView software version 5.5 (Bruker Corporation). For estimation of pial vessel diameter during functional activation, we used a 25× objective with N.A. 1, 4 mm working distance, and cover glass correction (XLPLN25XSVMP2, Olympus, Shinjuku, Japan). Excitation of the fluorophore was performed with 900 nm using a femtosecond laser (Chameleon Ultra, Coherent, Santa Clara, United States). Emission was detected by a Gallium-Arsenide-Phosphide photomultiplier (GaAsP-PMT, Hamamatsu, H10770PB-40) using a 660/40 nm-emission filter. Two kinds of acquisitions were performed:

1) T-series Scans for estimation of pial vessel diameter. Scans of 174 × 174 pixels (2.65 microns/pixel) were performed using spiral scanning mode to achieve a time resolution of ∼10 fps. Scans lasted 25 s, during which the contralateral whiskers were stimulated for 2 s with a custom-built air-puff stimulator. Estimation of pial vessel diameter was performed using ImageJ (NIH, Bethesda, MA, United States) and the macro VasoMetrics previously described in McDowell et al. (2021).
2) Angiograms of 500 μm (optimal z-resolution = 1.34 μm) and FOV of 462 μm^2^ (512 × 512 pixels) were acquired to visualize pial vessel topology. The angiogram was reconstructed with ImageJ and the 3D Viewer.

#### Functional activation

The paradigm for whisker stimulation to induce neuronal activity to measure neurovascular coupling effects in the sensorimotor cortex was epochs of 5 s pre-delay, 2 s stimulation (2 s of 3 Hz = 6 puffs), and 18 s post-delay. Stimulation is expected to increase blood flow, arterial blood volume, and oxygenation in venous blood (Boas and Dunn, 2010), providing measures detectable by OISI (blood volume by changes in Hb) and LSCI (rBFI). The mice were briefly trained with stimulation prior to recording to reduce jerks and sudden movements.

## Results

### *In vivo* magnetic resonance imaging

**FIGURE 5:**
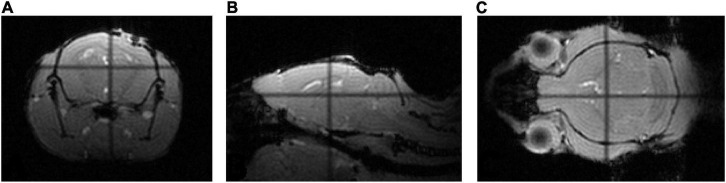
MRI localizer images. **(A–C)** Coronal, sagittal, and axial GE images acquired through a localizer scan show little to no distortion around the brain.

**FIGURE 6:**
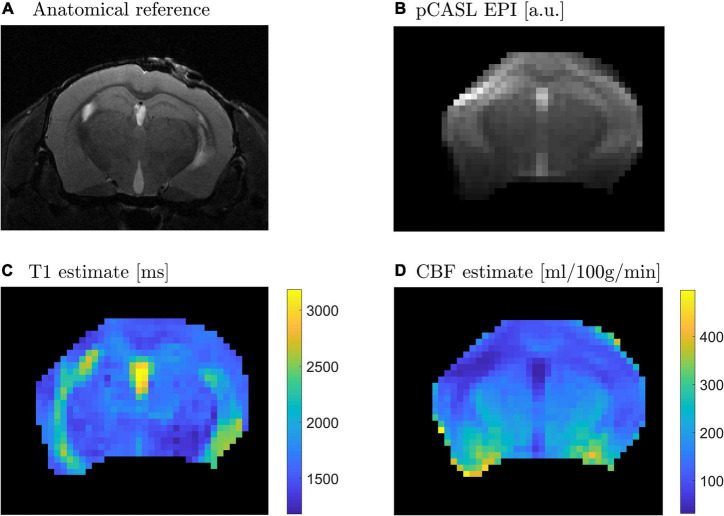
CBF parameter calculations. **(A)** Coronal slice as an anatomical reference; **(B)** EPI image acquired through a pCASL sequence shows only slight blurring along the surface of the brain; **(C)** Average inversion times in ms; **(D)** CBF map in ml/100 g tissue/min.

Sample pCASL and anatomical data show the satisfactory collection of MRI data using the described setup. The localizer scans, gradient echo (GE) anatomical images, are sensitive to differences in magnetic properties. The lack of distortion around the brain in all three slices (Figure 5) demonstrate the ability of the head holder and bed combination to remove movement as a factor to consider during scanning. Slight blurring along the surface of the brain is evident in the EPI image (Figure 6B) from the pCASL sequence. EPI sequences are also highly sensitive to magnetic susceptibility artifacts (Bitar et al., 2006), and therefore, since no extreme distortions are present, neither the head holder nor the window drastically affect image quality. Figure 6 further displays parameter calculations included in the quantification of CBF. The T1 map (C) shows average longitudinal relaxation times in ms used in the subsequent calculation of the CBF map (D) depicting perfusion in ml/100 g tissue/min. High T1 values are evident in the ventricles of the brain, and the highest CBF values are seen in the ventral part of the brain. pCASL is useful in quantifying CBF, though acquisition is not limited to the type of data shown here. Figures 5 and 6 depict a slight compression of the brain, likely attributed to the placement of the cranial window. This deformation of the cortex, however, does not affect the MR image quality which is seen to be free of distortions. Although the quality of cerebral hemodynamic data acquired here isn’t affected, this may be a limitation in other paradigms.

### *In vivo* optical intrinsic signal imaging/laser speckle contrast imaging

**FIGURE 7:**
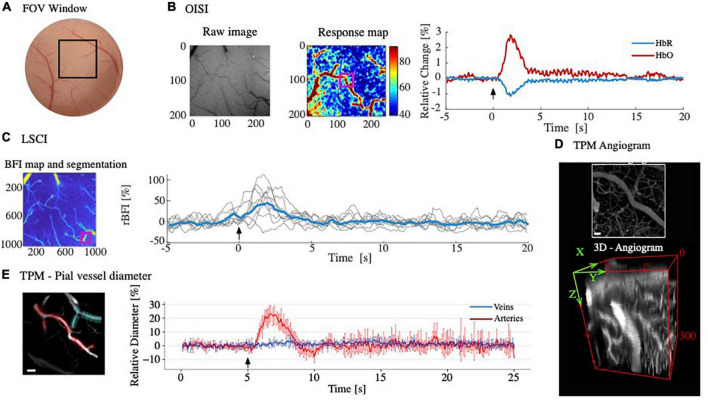
Acquired data from optical imaging. **(A)** Field of view with outlined region of interest used for OISI, LSCI, and TPM (black square); **(B)** OISI raw image (*left*) and response map (*middle*) as relative change from baseline (%). A region of interest (ROI; purple) was selected in the response map to plot relative changes as a function of time for HbR (blue) and HbO (red). The expected increase in HbO and decrease in HbR are seen as a result of stimulation (black arrow); **(C)** LSCI blood flow index (BFI) map and segmentation based on average contrast analysis from raw speckle signal. Relative BFI (rBFI) of an ROI (purple square) is plotted as a function of time from the intensity in each pixel of the signal and shows increased flow with stimulation (black arrow); **(D)** TPM maximum intensity projection and 3D angiogram used to visualize depth of imaging permitted with the cranial window; **(E)** TPM visualization of pial vessel diameters (*left*) and relative changes in pial vessel networks as a function of time (*right*). Estimations were performed using Vasometrics plugin in ImageJ, averaging all vessel branches from the arterial (red in *left*) and venous (cyan in *left)* networks selected from the TPM time-series. Upon activation (black arrow) arterial network showed an increase in diameter (red in *right*), whereas venous network was unresponsive to stimulation (blue in *left*). Scale bar = 50 μm.

The field of view (FOV) and imaged area of the brain are shown in Figure 7A. Figure 7B shows the OISI raw region of interest and response map for hemoglobin concentration and plots of relative intensity change as a function of time for derivatives of Hb (HbT and HbO) from the selected ROI. The expected stimulation-induced increase in HbO and decrease in HbR following vasodilation and increased blood flow to areas of high metabolic demand (9,17,18) were observed (Figure 7B). LCSI provides a measure of relative blood flow. Figure 7C presents a relative blood flow index (BFI) map and rBFI as a function of time. The increase in blood flow is evident as a result of vasodilation with functional activation.

### *In vivo* two-photon microscopy

Figure 7D depicts a maximum intensity projection and a tri-directional angiogram of the cortical vascular network. Angiograms can be used to segment vessels to achieve pial vessel morphometrics and capillary diameter and density. Pial vessels and their relative diameter change as a function of time are visualized and plotted in Figure 7E. We observed an expected dilation of the arterial tree during functional activation, whereas the venous networks remained unresponsive.

## Discussion

In this protocol, we proposed a methodology to integrate MRI and optical imaging techniques in awake-restrained mouse models with custom designed head holders and cradles that are easily reproducible. Surgical implantation of a head holder and cranial window combination for awake-restrained imaging in MRI-compatible beds enables multimodal imaging in the same subject, decreasing inter-subject variability (INCOSE, 2006) to achieve more representative data for interpretation of metrics of cerebral physiology from a wide range of methodologies (Devor et al., 2012). In addition, acquiring data from different imaging techniques in the same animal reduces the number of animals needed in experimentation following the current principle of the 3 Rs (Replacement, Reduction, and Refinement) recommended for animal research (MacArthur Clark, 2018).

For integrated imaging, head holders are required to be MRI-compatible and with long-term durability for serial imaging and handling. Materials and implantation must therefore ensure stability of the head and neck during scanning to avoid image distortions in both imaging setups. Sequential MRI and optical imaging in awake mice are enabled by a custom 3D printed head holder and bed combination for restraint and comfort. A successful alternative to rotating the head of the mouse for level imaging (Li et al., 2018) was introduced with a hole and metal rod insert in the optical imaging bed to avoid unnatural positioning of the head and neck. PETG plastic was used in the design and was found to be strong and durable enough to withstand movement of the body while keeping the head and neck stable. Stronger plastics may exist and improve the design, though these were not tested throughout the development of the design. Further, when housed together, mice were found to chew on the protruding parts of the frontal head piece of their cage mates. This could also be improved by printing with stronger plastic.

### Surgical considerations

Dried blood is a serious concern for signal loss and image distortion of MR images (Gaeta et al., 2021). Preventing and stopping bleeding from the brain, tissue, and skull during surgery is imperative regarding animal experimental ethics and good image quality. The quality of the windows could be assessed based on the extent of active bleeding from the dura mater, long-term transparency or obscurity of the windows, and detachment from the skull throughout experimentation and handling. It is important to take care in the pressure applied to the brain when placing the window to ensure the cortex isn’t notably compressed. To ensure proper sealing of the window, we find it highly important to remove any excess Kwik-Sil around the window for Loctite glue to adhere to the skull. Although Kwik-Sil provides increased prevention of bone regrowth and a barrier between the glass and the brain, it may obstruct imaging, in some cases, if air bubbles are present. Therefore, it can be decided by users of the protocol whether or not Kwik-Sil should be used. The consistency of Meliodent when applied to the skull should be determined by users, as we found it to be preference based. It is, however, crucial to ensure the mixture is kept on and around the skull. It is further advised that users follow security guidelines to avoid exposure to Meliodent powder. The collar, frontal head holder, and height of the dental acrylic build may result in less space for the objective depending on the width and working distance. This should be taken into consideration when placing and securing the head pieces.

### Applications of the design

We highly prioritize animal welfare in the protocol. Animal welfare is a crucial component in research, and animals allowed to express innate behavior without pain, fear, or distress are considered in a good state of welfare (Prescott and Lidster, 2017). Good animal welfare should be upheld to avoid suffering, and the relevance of results must be considered regarding the cost-benefit factor of using animal models to represent disease. Facial- and audio cues were used as indicators of pain and discomfort during habituation to restraint and scanning in the imaging environments. Socialization and training were implemented to reduce stress during handling and imaging, and animals were not exposed to unnecessary stressors. The awake-restrained imaging setup allows for the acquisition of imaging parameters from MRI and optical imaging. In this case, the blood flow index of brain parenchyma acquired from OISI/LSCI and changes in pial vessels acquired from TPM during functional activation can be used to interpret whole-brain pCASL estimates of CBF from MRI. A similar approach for multimodal imaging has been recently applied, demonstrating that capillary stalling events measured by TPM are associated with a reduction in whole-brain CBF (Cruz Hernández et al., 2019). The lack of significant artifacts in anatomical images indicates that the cranial window did not compromise the quality of images from MRI. The design is not limited to acquiring the types of data shown here and can be applied to many experimental designs. Introducing a sensory stimulus through air-puff allows for neurovascular coupling (NVC) investigations through blood flow measurements to achieve indirect and direct BOLD signals with LSCI/OISI and BOLD fMRI for a deeper understanding of blood flow regulation in different anatomical and functional areas of the brain.

Placement of the tail catheter further allows for injection of dyes, e.g., indicator-dilution techniques (Gutiérrez-Jiménez et al., 2016) and intravascular oxygen tension estimates (Sakadžić et al., 2010). This vascular access would also allow for administration of MRI contrast agents. The cranial window is ideal for longitudinal studies, remaining clear for up to 3 months and thus allowing for serial analysis and studies investigating changes in cerebral physiology over time. Additionally, for use in combination with a cranial window approaches such as half crystal skull or crystal skull (Kim et al., 2016), the anterior projection of the collar can be filed slightly to avoid extending onto the sutures of the occipital bone.

## Conclusion

Improving the interpretation of translatable methods such as MRI is becoming increasingly important as methods in preclinical neuroscience research develop and advance to explore subtle physiological mechanisms. We demonstrate the success of the design and surgical procedure in acquiring imaging parameters to adequately estimate measures of cerebral hemodynamics in MRI and optical imaging techniques in the same awake animal. The quality of images acquired from gradient echo (GE) and echo-planar imaging (EPI) was not affected by the surgical implants, verifying the usefulness of the holder and bed combination in eliminating movement in MRI. OISI results show expected changes in blood hemoglobin concentrations due to increased blood flow to stimulated areas in the brain, and stimulation/induced increase in relative blood flow is demonstrated with LSCI. Acquisition of diameter change and angiogram from TPM indicates the versatility of the window. Achieving multimodal data acquisition provides descriptive views of multiple aspects of physiological properties and reduces the number of animals needed in experimentation, thus decreasing inter-subject variability.

## Data availability statement

The original contributions presented in the study are included in the article/supplementary material, further inquiries can be directed to the corresponding authors.

## Ethics statement

The animal study was reviewed and approved by the Danish Animal Experiments Inspectorate.

## Author contributions

SHM and EGJ drafted and wrote the manuscript. Designs for restraint are accredited to TBL, VD, and SHM. Surgical preparations are accredited to BW, EGJ, and SHM. Methods/software for pCASL acquisition was provided by LH, JMW, and ELB. Imaging was performed by SHM, BW, EGJ, TBL, and BH. The pipeline and software for acquisition of LSCI were provided by DP, and data analysis for MRI and optics was performed by EGJ and BH. All authors contributed to revision of the manuscript.

## Footnotes

1
https://github.com/CFIN-High-Field-MRI
